# Vapor-phase-synthesized fluoroacrylate polymer thin films: thermal stability and structural properties

**DOI:** 10.3762/bjnano.8.95

**Published:** 2017-04-26

**Authors:** Paul Christian, Anna Maria Coclite

**Affiliations:** 1 Institute of Solid State Physics, Graz University of Technology, 8010 Graz, Austria

**Keywords:** EGDMA, iCVD, in situ, PFDA, spectroscopic ellipsometry, temperature dependent, X-ray diffraction

## Abstract

In this study, the thermal, chemical and structural stability of 1*H*,1*H*,2*H*,2*H*-perfluorodecyl acrylate polymers (p-PFDA) synthetized by initiated chemical vapor deposition (iCVD) were investigated. PFDA polymers are known for their interesting crystalline aggregation into a lamellar structure that induces super-hydrophobicity and oleophobicity. Nevertheless, when considering applications which involve chemical, mechanical and thermal stresses, it is important to know the limits under which the crystalline aggregation and the resulting polymer properties are stable. For this, chemical, morphological and structural properties upon multiple heating/cooling cycles were investigated both for linear PFDA polymers and for differently strong cross-linked alterations thereof. Heat treatment leaves the chemical composition of the linear PFDA polymers largely unchanged, while a more ordered crystalline structure with smoother morphology is observed. At the same time, the hydrophobicity and the integrity of the polymer deteriorate upon heating. The integrity and hydrophobicity of cross-linked p-PFDA films was preserved likely because of the lack of internal strain due to the coexistence of both crystalline and amorphous phases. The possibility to finely tune the degree of cross-linking can therefore expand the application portfolio in which PFDA polymers can be utilized.

## Introduction

Fluoropolymers, such as polytetrafluoroethylene, are interesting for a variety of different applications due to their low surface energy. The resultant hydrophobic and oleophobic surfaces are used as biocompatible surfaces [1], antifouling coatings [2], and as low dielectric constant materials [3] for microelectronics. Perfluoroacrylates are particularly appealing for such applications, as they combine the hydrophobic properties of the fluorinated pendant groups with easy processability due to an unsaturated acrylate group, allowing for mild processing conditions. One of the greatest limitations to the long-term stability of perfluoroacrylate-based technologies are mechanical, thermal and chemical stresses on the materials [4–7].

Some super-hydrophobic and oleophobic surfaces based on perfluoroacrylates were previously prepared by initiated chemical vapor deposition (iCVD) [8]. The iCVD technique allows polymerization of the fluorinated monomers, while the chemical structure of the precursor(s) remains intact. Therefore, ultrathin (<100 nm) perfluoropolymers can be easily deposited with high control over the chemistry [9] and crystalline structure [10] of the resulting coatings. Different from other thin film polymer deposition techniques, iCVD takes place in a completely dry environment, eliminating the tedious need of dissolving the fluoropolymers. In addition, it also allows the chemical structure of the monomers to be retained at high deposition rates, especially when compared with pulsed plasma deposition techniques [11]. The mechanism of polymerization by iCVD mirrors that of radical polymerization in solution [12]. An initiator molecule is thermally decomposed into radicals by a filament heated to 250–300 °C. The radicals of the initiator selectively react with the vinyl bonds of monomer species absorbed on the substrate, initiating the polymerization. For this, the substrate is typically held below 60 °C. Chain growth then proceeds on the substrate surface until terminated by another initiator radical or another initiator-monomer fragment.

**Figure 1:**
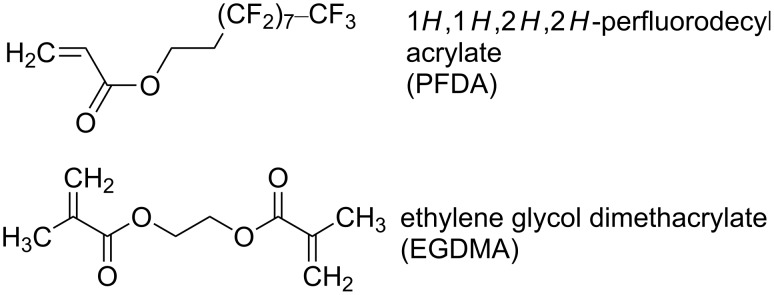
Molecular structure of the monomers PFDA and EGDMA.

The mechanical and chemical robustness of iCVD perfluoropolymers at elevated temperature has not yet been investigated. The aim of this study is to identify the limits of the thermal and mechanical stability of the 1*H*,1*H*,2*H*,2*H*-perfluorodecyl acrylate polymer (p-PFDA), and in order to enhance its durability, the copolymerization with ethylene glycol dimethacrylate (EGDMA) as a cross-linker is evaluated (the monomers are depicted in Figure 1). EGDMA is a diester with no free hydrophilic groups, which offers low viscosity, flexibility and high cross-link density in various polymer applications. Differently cross-linked p-PFDA films deposited by iCVD were previously studied, albeit with a somewhat different scope [13]. In contrast to the other cross-linkers that have been studied in combination with perfluropolymers (e.g., divinylbenzene (DVB)), EGDMA has a higher conversion rate, which results in a very low percentage of unreacted vinyl bonds after the deposition. It has been demonstrated that only after annealing, the perfluorinated films were strongly cross-linked by DVB resulting in films that showed low hysteresis between advancing and receding contact angle [14]. The advantage of cross-linking with EGDMA is the elimination of the annealing step, resulting in a cross-linked film already in the as-deposited form. The thermal stability of the thin films was evaluated by ellipsometry, Fourier transform infrared spectroscopy and X-ray diffraction. In situ ellipsometric studies allow the monitoring of the evolution of the thickness and optical constants of the materials during the heating ramp. This is particularly suitable for evidencing thermal transitions in thin films with thickness ranging from a few micrometers to monolayers [15–16].

## Experimental

The monomer precursor 1*H*,1*H*,2*H*,2*H*-perfluorodecyl acrylate (PFDA, purity 97%), the cross-linking agent ethylene glycol dimethacrylate (EGDMA, purity 98%) and the initiator *tert*-butyl peroxide (TBPO, purity 98%) were purchased from Sigma-Aldrich (Germany) and used without further purification. p-PFDA films with different degrees of cross-linking were prepared by initiated chemical vapor deposition (iCVD). The average thickness of the as-deposited polymer films was 350 ± 50 nm. Detailed information on the actual processing conditions are provided in Supporting Information File 1, while a full description of the setup can be found in a previous publication [17]. As substrates, silicon wafers with a native oxide layer (thickness 1.7 nm) were used after being cut into 2 × 2 cm^2^ pieces.

Fourier transform infrared spectroscopy (FTIR) was performed on a Bruker IFS 66v/s spectrometer in transmission mode, with all the data being converted to absorption spectra by the OPUS software. The data are automatically baseline corrected by a custom routine written in R, utilizing the algorithms provided in the baseline package [18].

Specular X-ray diffraction (XRD) patterns were recorded on a PANalytical Empyrean diffractometer. The system is equipped with a copper sealed tube (λ = 0.154 nm), a Göbbel mirror, various slits and a PIXcel^3D^ solid state detector. All data were recorded using the same setup and are represented in the scattering vector (*q_z_*) notation, whereby *q_z_ =* 4π·sin(θ)/λ*.* The index *z* indicates that only net planes parallel to the substrate surface were probed in the experiment (specular scan). In situ, temperature-dependent XRD studies were performed with a DHS900 heating stage attachment (Anton-Paar, Austria), using a heating rate of 2 °C/min. The integration time was set to one minute, meaning that a temperature resolution of 2 °C could be achieved.

Atomic force micrographs were taken in noncontact mode on a Nanosurf easyScan 2, equipped with a PPP-NCLR-10 cantilever (NanoWorld AG, Switzerland). The data are corrected for artifacts with the freely available software package Gwyddion [19].

The water contact angle (WCA) of the polymer films was determined by the static sessile drop method on a CAM200 contact angle analyzer (KSV Instruments, Finland). Each sample was probed on five different spots, using a droplet volume of 4 μL.

In situ temperature-dependent spectroscopic ellipsometry measurements were performed on a Woollam M-2000 ellipsometer (J.A. Woollam Co., USA), equipped with a THMS600 temperature stage (Linkam, UK) under nitrogen atmosphere. The samples were investigated in the temperature range between 10 and 150 °C at a heating/cooling rate of 5 °C/min, with a hold time of 5 min between steps. Prior to the measurement, the samples were equilibrated by three subsequent heating and cooling cycles. The optical data were recorded every second at an incidence angle of 75° in the wavelength range of 370 to 1000 nm. The ellipsometric data were modeled using the CompleteEASE^®^ software by a three-layer system consisting of the silicon substrate, the interfacial oxide layer and the transparent top layer. The wavelength- and temperature-dependent refractive indices of silicon and oxide were taken from literature [20], whereas Cauchy’s equation was utilized in modeling the polymer film. A nonlinear least squares fit of the experimental data with this model yields the optical constants and thickness of the polymer layers.

## Results and Discussion

### Chemical composition

**Figure 2:**
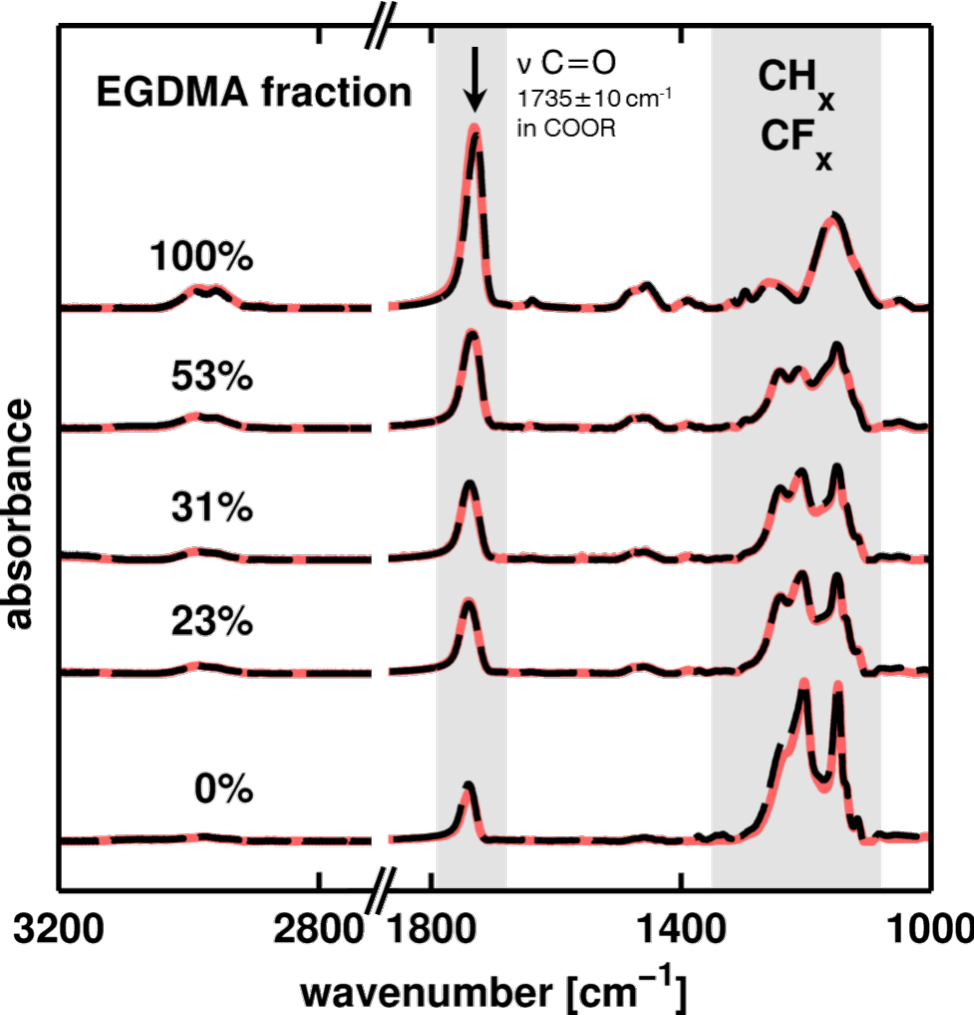
FTIR spectra of p-PFDA films with different EGDMA cross-linker ratios in the as-prepared state (dashed line) and after heat-treatment (solid line). The signal intensity is normalized by the film thickness. Data are shifted on the *y*-axis for clarity.

The retention of chemical functionality and the degree of cross-linking for the different samples were evaluated by FTIR spectroscopy. In Figure 2, the spectra of the as-prepared samples are depicted (solid lines), where the data are normalized with the polymer layer thickness (i.e., with the sampled volume). Starting with the spectrum of p-PFDA, several characteristic absorption peaks are noted. In the fingerprint region (1500–500 cm^−1^), the skeletal vibrations of the CH*_x_* and CF*_x_* groups are visible, most prominently featuring the symmetric and antisymmetric stretch of the CF_2_ groups at 1251 and 1206 cm^−1^, respectively. In addition, a strong absorption peak is observed at 1740 cm^−1^, stemming from the C=O stretching of the ester groups. Upon cross-linking, this peak increases in intensity as EGDMA has twice the number of C=O groups relative to PFDA. In the fingerprint region, the signal of the CF*_x_* groups decreases with increased cross-linking, eventually resulting in two distinct peaks of C–O stretching at 1257 and 1158 cm^−1^ for p-EGDMA. Additional peaks in the regions of 1480–1450 cm^−1^ and 3000–2800 cm^−1^ in the spectra of the cross-linked polymers are attributed to deformation and stretching vibrations of the CH*_x_* groups, respectively. Interestingly, a small peak at 1638 cm^−1^ is noted to appear exclusively for p-EGDMA, which is characteristic for C=C stretching [21]. This implies that the polymerization of the cross-linker is not completely facilitated as some unreacted vinyl bonds remained in the polymer. Nevertheless, this peak is absent for all other spectra, thus the fraction of unreacted monomer species (in the limit of the experiment) is likely small. This means that the postdeposition annealing steps are superfluous, which is a clear advantage over other cross-linkers such as divinylbenzene; for the latter, iCVD copolymerization with PFDA resulted only in minor conversion rates, necessitating a time-consuming thermal conversion after deposition [14].

To evaluate the degree of cross-linking, each spectrum is treated as a linear combination of the spectra of the p-EGDMA and PFDA homopolymers, each weighted with a factor accounting for its fraction. By a linear fit of these factors, the composition is then obtained (see labels of the spectra in Figure 2).

After repeated heating of such samples to 150 °C under nitrogen atmosphere, the chemical composition of the polymers remained unchanged (dashed lines in Figure 2). This means that both p-PFDA and cross-linked alterations thereof are chemically stable in the investigated temperature range, within the detection limits of the FTIR spectroscopy measurement.

### Surface morphology and wettability

**Figure 3:**
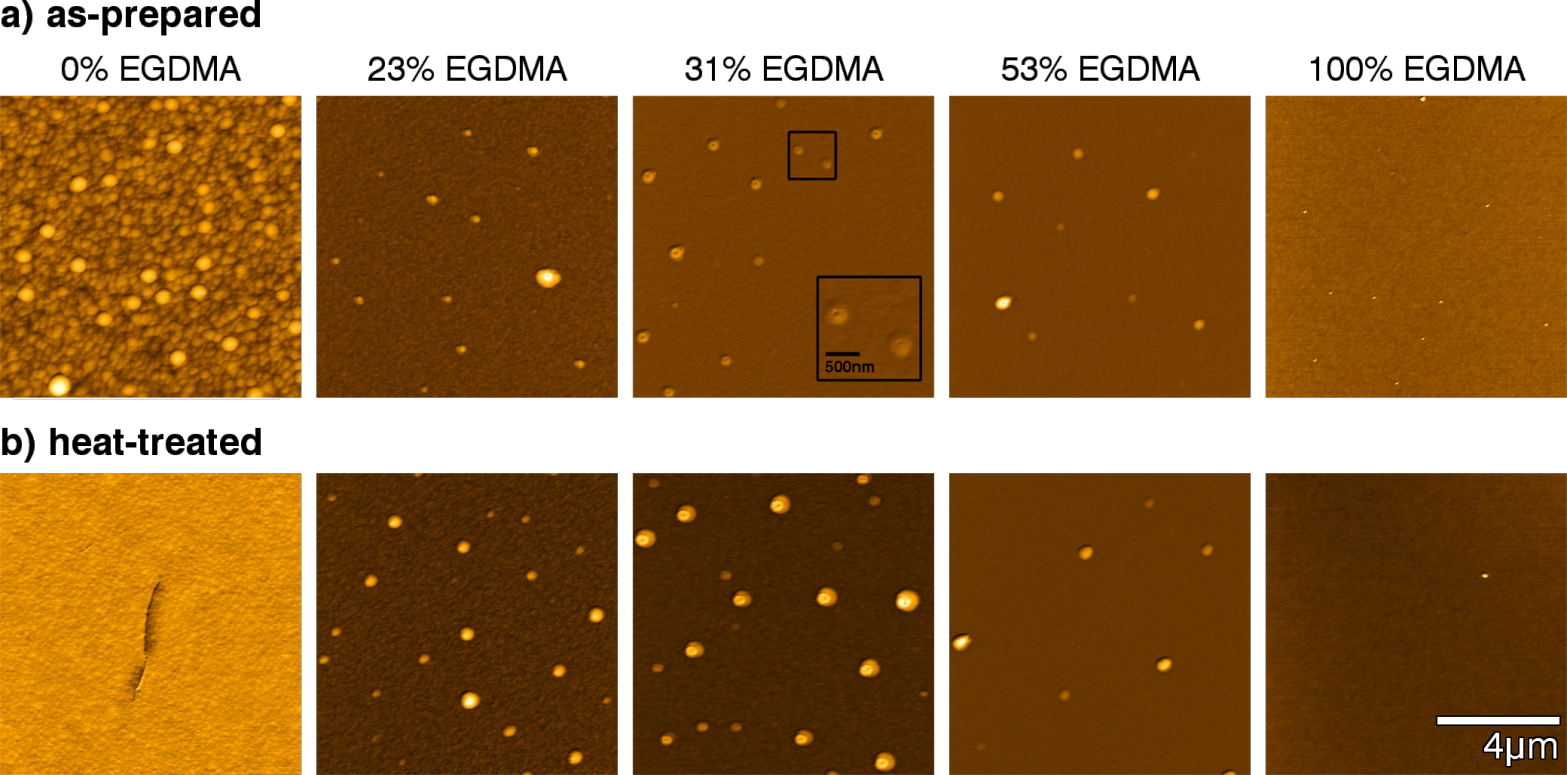
AFM height micrographs of as-prepared (a) and heat-treated (b) p-PFDA films with different degrees of EGDMA cross-linking. The data are represented on individual color scales for clarity.

The impact of the cross-linker on the surface morphology of p-PFDA films was investigated by atomic force microscopy (AFM) for the as-prepared and heat-treated samples (see Figure 3). For pristine p-PFDA films, the surface consists of randomly distributed spherical aggregations, forming a hillock-like structure. This is also reflected by the root mean square roughness (σ) of the surface, which was 23.7 nm. The mean radius of the spherical structures is about 200 nm, as determined from the autocorrelation length of the micrograph.

Upon addition of EGDMA, the surface morphology is drastically changed. The hillock-like structure of pure p-PFDA is reduced to a few aggregated clusters, interrupting the otherwise smooth polymer film. Except for these grains, the layer’s root mean square roughness is below 2 nm, indicating a rather smooth coating of the silicon wafer (roughness below 1 nm). This morphology persists as the EGDMA content increases, suggesting that the transition from spherical aggregations to a smooth coating occurs already at small additions of cross-linker. For the p-EGDMA film, no surface aggregations are observed and a smooth coating results (roughness below 1 nm). The absence of spherical structures in p-EGDMA and the close resemblance to the morphology of p-PFDA films suggest that these aggregations are in fact just due to the PFDA units, as shown also in previous works [10,14]. An interesting detail is observed in the AFM scan of a sample containing 31% EGDMA (third image from left in Figure 3a). Small notches are noted for all the grains, extending twenty to thirty nanometers into the film (a more detailed view is shown in the inset). Possibly, these structures are the result of the degassing of unpolymerized monomer units, which are “buried” within the film during deposition. However, this behavior does not occur for the other samples (or is at least not observable in the respective AFM scans) and is uncharacteristic for iCVD polymers in general. Therefore, it is assumed that such structures are specific for this very sample and not characteristic for this particular EGDMA concentration.

After repeated heating of these polymer films to 150 °C, changes in surface morphology were recorded by AFM (Figure 3b). For the p-PFDA films, a completely different surface morphology results. The spherical aggregates in the as-prepared polymer are completely absent and a relatively smooth surface results instead. The morphology is still reminiscent of the hillock-like structures but on a much smaller scale; the roughness decreases to below 2 nm, comparable to that of cross-linked surfaces. However, multiple cracks have formed in the polymer film, extending several micrometers in the lateral direction (an example is shown in Figure 3, see Supporting Information File 1, Figure S1 for larger scales). The line profiles of these cracks reveal a penetration depth of approximately 20 nm, which means that they are limited to the interfacial area (the film thickness is approximately 350 nm). For cross-linked films, the temperature treatment results in no observable changes, indicating good stability towards temperature variation in the investigated range.

**Figure 4:**
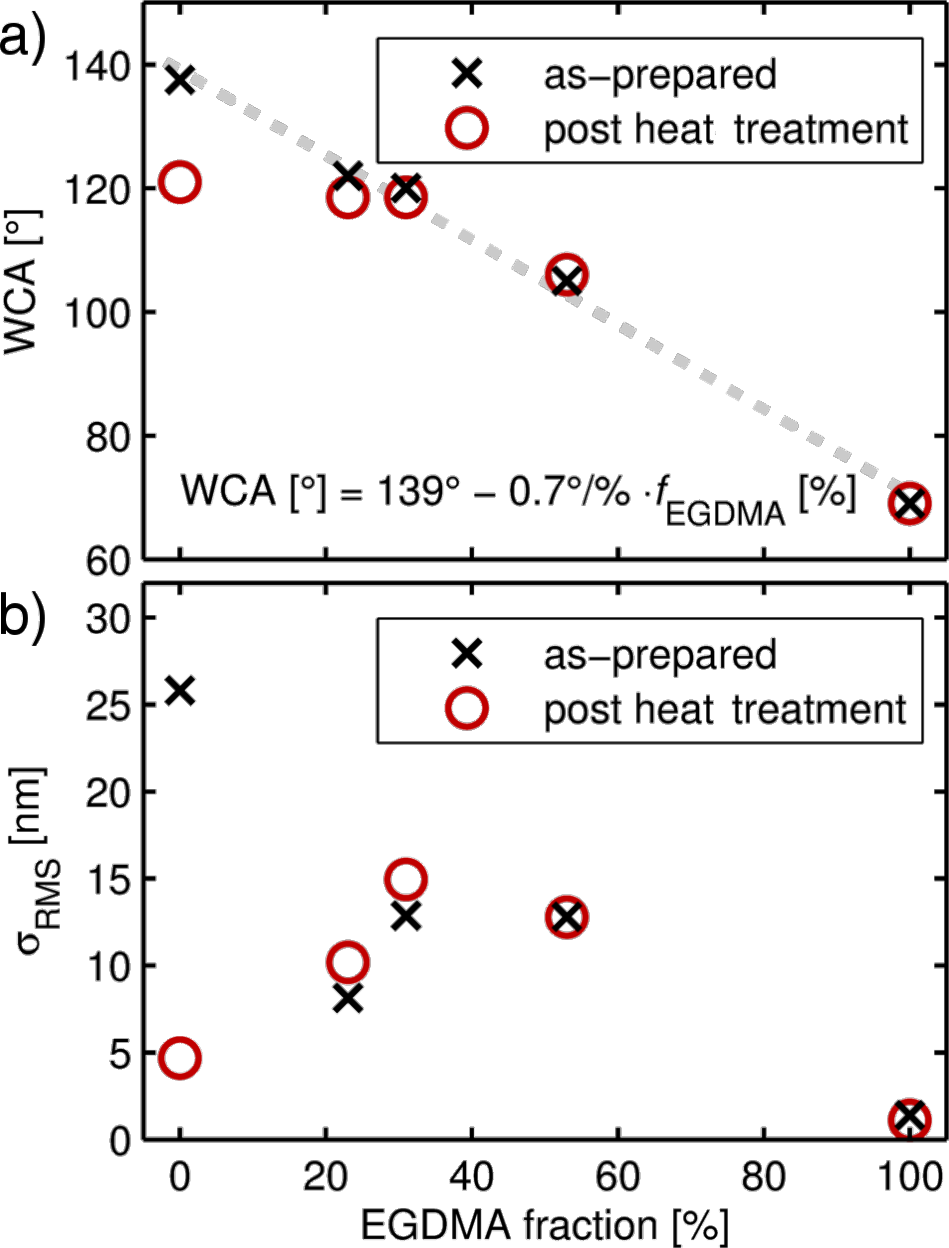
Water contact angle (WCA) (a) and root mean square surface roughness (σ_RMS_) (b) of p-PFDA films with different degrees of EGDMA cross-linking, as determined by the static sessile drop method and from AFM, respectively. The σ_RMS_ values were calculated from the 50 × 50 μm² AFM micrograph areas presented in Supporting Information File 1, Figure S1. For the as-prepared films, a linear relationship between the contact angle and the cross-linker ratio is found (dashed line). Error bars have been omitted for clarity as the standard deviation of the WCA was less than two degrees.

Changes in the surface morphology and chemistry also affect the wettability, as evidenced by a decreasing water contact angle (WCA) upon EGDMA addition to the polymer (Figure 4a). While the PFDA homopolymer forms a highly hydrophobic surface with a WCA of 138 ± 2°, a linear decrease results as the fraction of EGDMA cross-linker is gradually increased. The intercept with the *y*-axis was one of the fit parameters and the fit result, 139°, falls within the error range of the physical measurement of the water contact angle of the PFDA homopolymer. For the EGDMA homopolymer, a WCA of 69 ± 1° was ultimately observed. The change in wettability stems from the (relative) increase of carbonyl groups upon addition of EGDMA, which turns the polymer more hydrophilic. Similar to the morphological changes discussed above, heat treatment predominantly affects the water contact angle of the PFDA homopolymer. A decrease of the WCA to 121 ± 1° results, while the cross-linked polymers show little to no change, independent of the degree of cross-linking.

In Figure 4b, the root mean square surface roughness, σ_RMS_, of the full-scale AFM micrographs (Supporting Information File 1, Figure S1, 50 × 50 μm^2^) is plotted as a function of EGDMA content, both for the as-prepared and heat-treated films. The data evidence that heat treatment predominantly affects the PFDA homopolymer, while only minor changes are noted for cross-linked alterations. Despite the formation of cracks, a pronounced decrease in the σ_RMS_ value was observed for the PFDA homopolymer, resulting in a much smoother film. This suggests that the initial WCA of the p-PFDA films stems in fact from a combination of hydrophobicity by the perfluorinated groups and the high surface roughness present in the as-prepared films. On the other hand, the effect of elevated temperature shows no or only a negligible effect on the morphology and chemical composition of cross-linked polymers (in the investigated range). This means that after deposition, these compositions are already closer to equilibrium so that less internal strain occurs. In turn, this makes these films less prone to rupture formation or surface rearrangement upon exposure to elevated temperatures. The data also show that in thermally annealed films, a water contact angle of approximately 120° can be maintained up to EGDMA fractions of 40%, indicating a surface composition dominated by the fluorinated groups of the PDFA portion.

### Structural characterization

**Figure 5:**
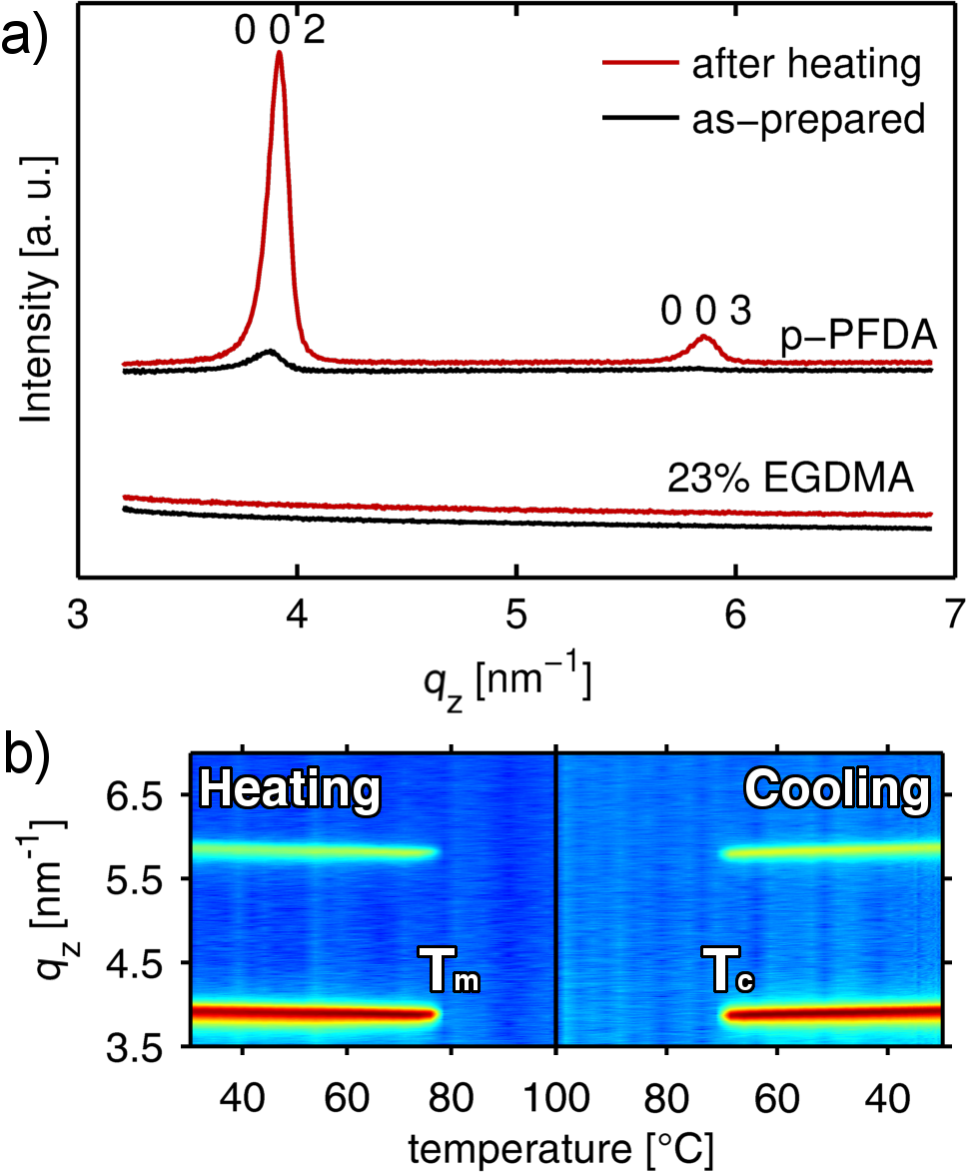
(a) Specular X-ray diffraction patterns of a p-PFDA film and a cross-linked alteration thereof with 23% EGDMA content in the as-prepared state and after heating to 150 °C. For clarity, data are shifted along the *y*-axis (intensity). (b) Temperature-dependent in situ X-ray diffraction measurements of crystalline p-PFDA at a rate of 2 °C/min, with *T*_m_ and *T*_c_ denoting the onset of melting and of crystallisation at 76 and 69 °C, respectively.

The distinct features of pure p-PFDA films and cross-linked alterations thereof are not only limited to the interface but are rather the result of differences in the bulk of the thin films. This is evident from the specular X-ray diffraction data of the as-prepared films, as depicted in Figure 5a. Pristine p-PFDA exhibits a low intensity peak at *q_z_* = 3.88 nm^−1^, meaning that this film is (at least partially) crystalline. This peak is characteristic for the hexagonal packing of the fluorinated pendant chains into a bilayer structure of distance *d* = 3.18 ± 0.02 nm (corresponding to a scattering vector *q_z_* = 1.98 nm^−1^) [4]. Thus, the Bragg peak in Figure 5a is identified as the 002 reflection of this lamellar packing (data depicting the 001 reflection is provided in Supporting Information File 1, Figure S2). Additionally, the data show a preferred orientation of the lamella that is parallel to the substrate surface, as other reflections are absent in the spectra (measured up to *q_z_* = 20 nm^−1^).

A very different behavior results as the PFDA polymer is cross-linked by the addition of EGDMA to the iCVD process. The crystalline features of the PFDA homopolymer are absent in the cross-linked alterations, independent of the tested EGDMA fraction (thus, only the sample with the lowest EGDMA content is shown). The cross-linked polymers lack a defined repeating unit due to the random nature of the chain interconnects, which constitute a steric hindrance for chain rearrangement, thus limiting the formation of any long-range order.

The structural difference between the PFDA homopolymer and its cross-linked alterations most likely accounts for their distinct thermal response in terms of morphology (see the AFM data in Figure 3), while the chemistry remains unaffected by the heat treatment (see the FTIR data in Figure 2). After heating to 150 °C and cooling back to ambient, the cross-linked polymers do not show any change in the XRD pattern, while the 002 Bragg reflection of the PFDA homopolymer displays a strong increase in intensity. In addition, a further (higher order) reflection of the lamellar structure emerges at *q_z_* = 5.86 nm^−1^, corresponding to the 003 plane. This behavior suggests that thermal treatment greatly improves crystallinity and/or mosaicity of the PFDA homopolymer, but does not lead to the formation of crystalline domains in the cross-linked films. Likely, with a lower cross-linker fraction, some degree of crystallinity might be preserved.

To gain further insight into the thermally induced structural changes of p-PFDA films, in situ X-ray diffraction experiments were performed. In Figure 5b, the specular diffraction pattern of the PFDA homopolymer is depicted in a pseudo-color representation as a function of temperature. The data features most prominently the positions of the 002 and 003 Bragg reflections, which display a shift towards lower *q*-values (and thus larger lattice distances) upon temperature increase, corresponding to thermal expansion of the unit cell. At 76 ± 2 °C, a sudden decrease in the diffracted intensity is then observed, denoting the melting point of the lamella. As the temperature is further increased to 100 °C, no change is observed in the diffraction pattern, that is, the polymer remains in the amorphous state. Upon cooling, recrystallization occurs at 69 ± 2 °C. Once they have emerged, these Bragg peaks display little variation in intensity as a function of temperature, suggesting that the polymer side-chains assume the final lamella arrangement within the resolution of the experiment (2 °C/min). On the other hand, a more pronounced shift of the Bragg peak positions towards higher *q*-values is noted upon cooling.

To quantify the thermal expansion/contraction of the p-PFDA unit cell, the coefficients of linear thermal expansion (CTEs) both for heating (α_heating_) and cooling (α_cooling_) are determined from the data. In general, the CTE is defined as

[1]
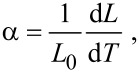


with d*L*/d*T* denoting the rate of change in thickness with temperature, normalized by an initial thickness *L*_0_ (for this work, *L*_0_ refers to the length at 25 °C). From a linear fit to the data in Figure 5b, the linear CTEs are determined to be α_heating_ = (2.18 ± 0.05) × 10^−4^ K^−1^ and α_cooling_ = (3.2 ± 0.1) × 10^−4^ K^−1^. The difference between the CTEs of the heating and the cooling run indicates that the sample has not yet reached an equilibrium state, which is expected for an as-prepared sample. Upon recrystallization under cooling, the Bragg peaks are slightly shifted towards higher *q*-values (see Figure 5a,b); the bilayer distance is reduced with respect to the as-prepared state and minor relaxation has occurred in the p-PFDA unit cell. While this difference diminishes in subsequent runs, an increasing peak intensity is still observed after the third run (data not shown).

**Figure 6:**
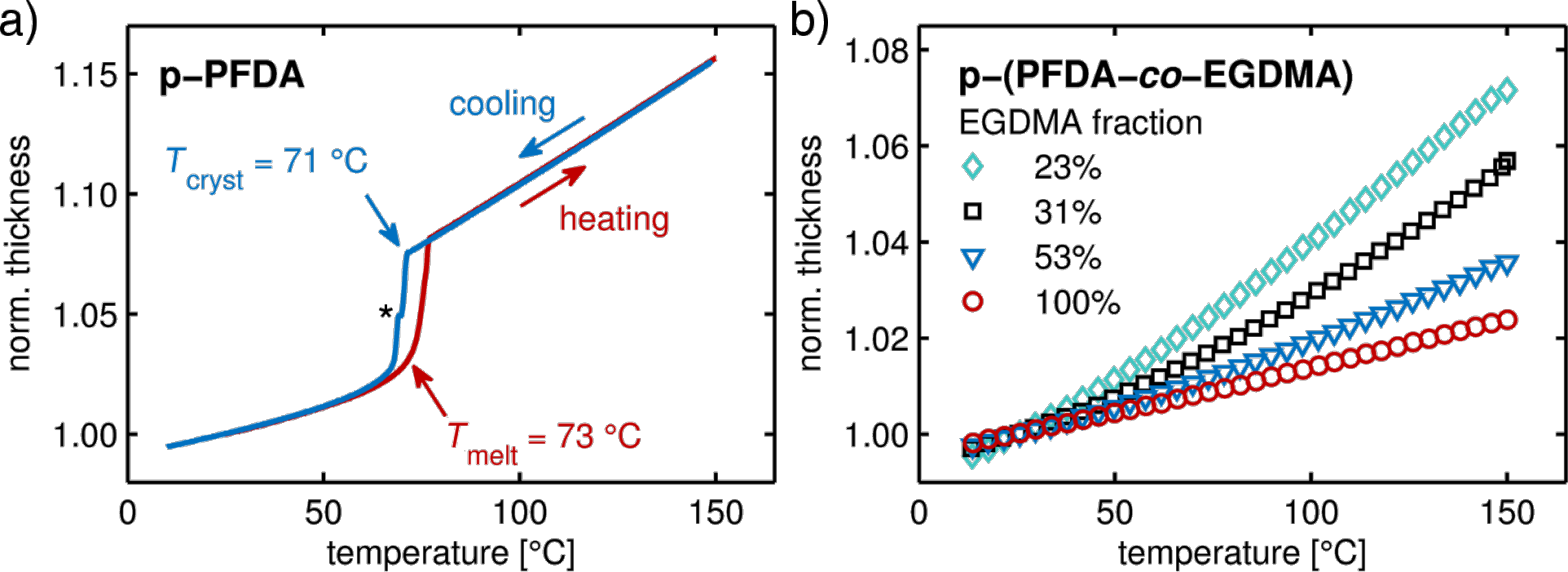
In situ spectroscopic ellipsometry data depicting film thickness evolution as a function of temperature for a PFDA homopolymer (a) and for samples containing different degrees of EGDMA cross-linking (b). The heating/cooling rates were 5 °C/min and data are normalized to the thickness at 25 °C. The data in (b) has been reduced for clarity and only cooling runs are shown. Please note that the discontinuity in (a) is an artefact and has been marked by an asterisk.

While X-ray diffraction techniques are perfectly suited to follow structural processes in crystalline materials, their application to amorphous materials is less favorable. To also provide some insight into the amorphous, cross-linked p-(PFDA-*co*-EGDMA) films, in situ spectroscopic ellipsometry was utilized. In Figure 6, the evolution of the film thickness (normalized to the thickness at 25 °C) is depicted as a function of temperature, as determined from ellipsometry data. After several equilibration cycles (as described in the Experimental section), a reversible behavior is recorded. Figure 6a shows a typical measurement for a PFDA homopolymer for a heating and a cooling run. The data features most notably a first-order phase transition, with the onsets at 73 and 71 °C, determined by linear fits to the data. These points correspond to melting, and respectively, crystallization of the lamella. These thermal transition points are in reasonable agreement with those determined from the X-ray diffraction experiment (76 ± 2 and 69 ± 2 °C). The difference is attributed to general sensitivity and temperature resolution differences between these two techniques. While in the X-ray diffraction experiment a resolution of only 2 °C is achieved, the ellipsometric measurement features a ten-times better temperature resolution. In addition, ellipsometry allows even very minor changes in film thickness to be monitored without the need of long integration times.

Above and below the transition point, the data features the thermal expansion of the p-PFDA film. The observed changes do not depend on whether the experiment is performed while heating or cooling, hinting at a reversible behavior. This also means that no thickness loss occurs during the experiment. As this behavior was noted for all the samples (within this temperature regime), only cooling runs are considered from here on.

When EGDMA is added to the polymer, a different behavior was revealed (Figure 6b). A strong thermal transition, as observed for the PDFA homopolymer, is absent. This is expected as X-ray diffraction scans did not show any crystalline fraction (Figure 5a). Instead, the data evidence a gradual decrease in the thermal expansion as the EGDMA content increases. In addition, the thermal expansion features a slight curvature, indicating a thermal transition.

**Figure 7:**
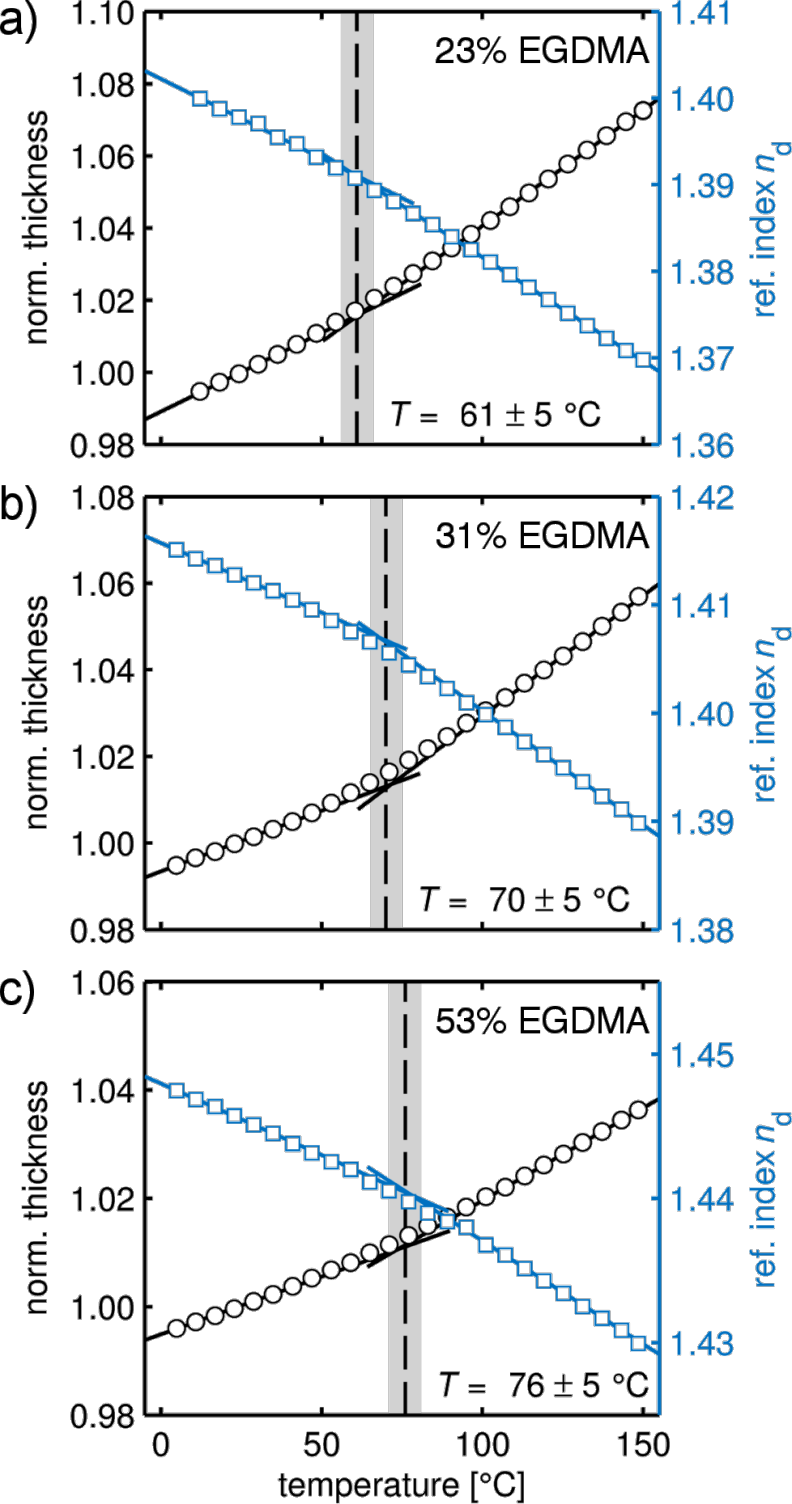
Normalized film thickness and the refractive index *n*_d_ (at λ = 589.3 nm) as a function of the temperature for p-(PFDA-*co*-EGDMA) films with (a) 23% EGDMA, (b) 31% EGDMA, and (c) 53% EGDMA content. From the intersection of the linear functions fitted to the data, the thermal transitions are evidenced. The corresponding transition temperatures are annotated in the graph. The shaded area represents the error bar.

To investigate this in more detail, changes in film thickness and in refractive index are depicted together in Figure 7 as a function of temperature. For a EGDMA fraction of 23% (Figure 7a), a thermal transition at *T* = 61 ± 5 °C is evidenced by the intersection of two linear fits to the data. With an increasing EGDMA content, this thermal transition shifts to higher temperatures but also becomes less pronounced (Figure 7b,c). Finally, for the EGDMA homopolymer, no transition is observable. While the thermal expansion decreases with increasing EGDMA content, the opposite behavior is noted in the refractive index. Interestingly, this shift in the refractive index (at ambient temperature) shows a linear behavior with the EGDMA fraction and could thus be used to determine the copolymer ratio. The obtained ratios are in good agreement with those determined from FTIR measurements (within a few percent). It is worth noting that another feasible way to evidence such transitions is to determine the thermal expansion coefficient at each temperature by numerical differentiation [22]. This procedure results in comparable transitions points (data not shown) but is very sensitive to noise in the fit/measurement. Thus, measurements should then be performed under stationary (isothermal) conditions for each temperature.

The origin of the thermal transitions could not be unambiguously identified. While crystalline fractions and first-order phase transitions were (in the limit of the experiments) not evident in the present data, the position of the thermal transitions suggests a relation to the crystalline packing present in the PFDA homopolymer. Likely, the thermal transition originates from a collective movement of the perfluorinated PFDA sidechains upon temperature increase. Compared to the liquid–crystalline state, the energy barrier for such chain movement is lowered as lattice energy is absent. This is in agreement with the sample containing 23% EGDMA, which exhibited the lowest transition point at *T* = 61 ± 5 °C. With stronger cross-linking, the mobility of the fluoroalkyl groups is lowered, thus yielding a shift to higher temperatures while also yielding weaker transitions in general.

There are also several reports in literature which mention a glass transition in the EGDMA homopolymer, located between 130–140 °C [23–24]. However, such a transition is not evident in the present data. While thin films typically do show a different behavior compared to bulk materials, a film thickness greater than 100 nm (such as the ones used in this study) is often found in agreement with the bulk [25–26].

**Figure 8:**
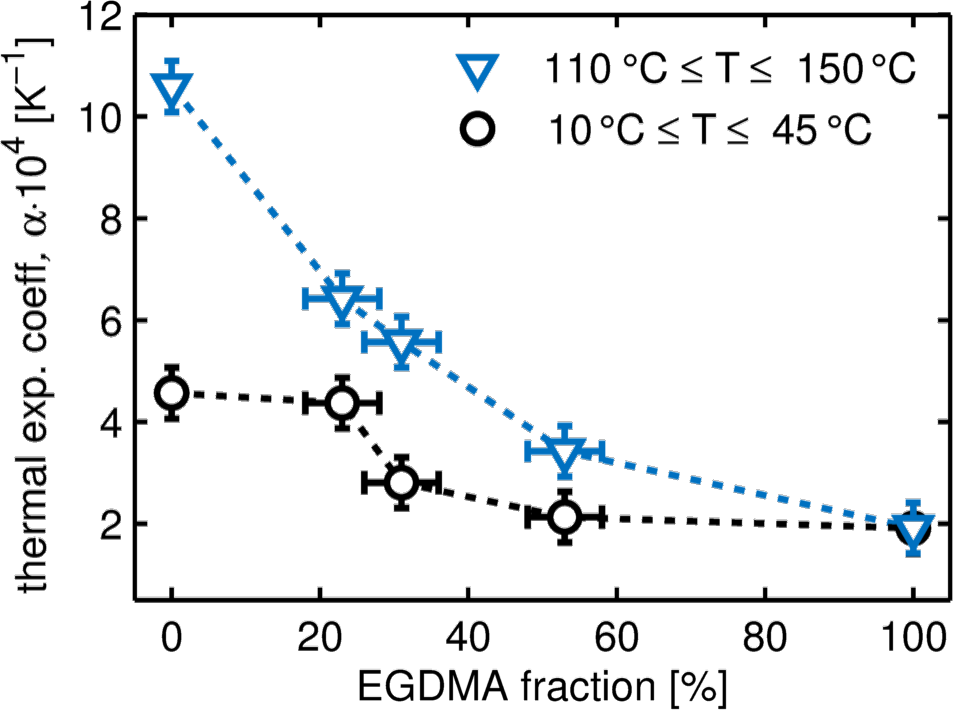
Coefficient of linear thermal expansion, α, as a function of EGDMA cross-linker fraction for various p-(PFDA-*co*-EGDMA) films in two temperature regions. The data points are interconnected by dashed lines as a guide to the eye.

In addition, CTEs are determined for regions in which changes in film thickness depend linearly on temperature. Figure 8 displays the change in thermal expansion coefficients as a function of EGDMA content in the regions below and above the thermal transitions points ((10–45 °C) and 110–150 °C), respectively). Below the melting point, the PFDA homopolymer exhibits the highest thermal expansion coefficient of the systems studied. A comparison with the thermal expansion of the crystallites shows that the thermal expansion of the lamella (α_cryst_ = 2.18 ± 0.05 × 10^−4^ K^−1^) accounts only for about half of the total film thickness increase (α_film_ = 4.5 ± 0.5 × 10^−4^ K^−1^). This suggests that the disordered regions between the layers strongly impact the thermal properties. Possibly, this mixture of amorphous and liquid–crystalline film portions leads to a thermal mismatch, causing the rupture formation noticed within the film. Above 110 °C, the thermal expansion coefficient of p-PFDA is drastically increased as the melting point of the lamella is surpassed (α_melt_ = 10.6 ± 0.5 × 10^−4^ K^−1^).

As EGDMA cross-linker is added to the polymer, the CTEs decrease and the difference between CTEs above and below the thermal transition also decreases until it is fully absent for the p-EGDMA homopolymer. This behavior reflects the fact that thermal transitions become weaker with increasing EGDMA content and are fully absent for the p-EGDMA homopolymer (in the investigated temperature range). As the WCA (i.e., the surface energy) displayed little dependence on the EGDMA content (below 40% fraction), this allows for the deposition of highly hydrophobic p-(PFDA*-co*-EGDMA) surfaces with control over the thermal expansion (and mechanical properties) being established by the cross-linker degree.

## Conclusion

The morphological and structural properties of linear and cross-linked p-PFDA films deposited by iCVD were investigated with the aim of identifying the limit of thermal stress that these films can sustain before losing integrity. PFDA polymers have indeed shown very interesting properties in terms of repellence of oil and water, due to the formation of a crystalline lamellar structure between the PFDA chains. While this makes the p-PFDA highly suitable for a large variety of applications, it is interesting to know how these properties change with temperature oscillations that can occur during the everyday use of technologies based on this polymer.

The present study shows that the mechanical stability can be greatly improved by the addition of a cross-linker. When the linear p-PFDA was exposed to the heating cycles, the chemistry remained unchanged while the crystallinity of the films was largely improved and the morphological character of the surface became smoother. Nevertheless, the thermal stress caused some ruptures in the films and reduced the hydrophobic properties. EGDMA, added as a cross-linker, was shown to preserve the chemical stability and hydrophobicity of p-PFDA coatings while making the film more cohesive. The loss of the structural integrity in the PFDA homopolymer was attributed to the different thermal expansion coefficients of the crystalline and amorphous film portions, which caused tension in these films upon heating/cooling. The cross-linked films were fully amorphous, also upon heating, but had more stable hydrophobic properties and showed an increased crack resistance.

In addition, this study demonstrated that EGDMA is a feasible cross-linker for the synthesis of thermally stable hydrophobic polymers. While the ester groups can become a limiting factor at even higher temperatures, EGDMA offers unique advantages for applications below 150 °C. It outperforms previously employed reagents like DVB, allowing for faster deposition rates in the iCVD process and making postdeposition curing unnecessary, as high conversion rates are readily achieved.

The chosen deposition technique, iCVD, allows fine-tuning of the cross-linking ratio, different from other vapor-based deposition techniques (e.g., plasma-enhanced CVD). Therefore, depending on the application and on the desired polymer properties, one can choose to work in conditions that drive crystallinity and hydrophobicity or thermally stable surface properties instead.

## Supporting Information

File 1 Additional experimental parameters and results.
iCVD process parameters used in the sample deposition; atomic force micrographs depicting larger scales; X-ray reflectivity scan evidencing the bilayer structure of p-PFDA.
