# Supplementary material for: Vapor-phase-synthesized fluoroacrylate polymer thin films: thermal stability and structural properties
Source: Beilstein J Nanotechnol. 2017 Apr 26;8:933–42. doi: 10.3762/bjnano.8.95 (PMC5433210; doi:10.3762/bjnano.8.95)
Supplement: File 1 — Additional experimental parameters and results. iCVD process parameters used in the sample deposition; atomic force micrographs depicting larger scales; X-ray reflectivity scan evidencing the bilayer structure of p-PFDA. [file Beilstein_J_Nanotechnol-08-933-s001.pdf]

Supporting Information

for

Vapor-phase-synthesized fluoroacrylate polymer thin films: thermal stability and structural properties

Paul Christian[1] and Anna Maria Coclite[1,*]

Address: [1]Institute of Solid State Physics, Graz University of Technology, 8010 Graz, Austria

Email: Anna Maria Coclite* - anna.coclite@tugraz.at

*Corresponding author

Additional experimental parameters and results

iCVD deposition conditions

The following parameters were used in the synthesis of 1H,1H,2H,2H-Perfluorodecyl acrylate (PFDA) and ethylene glycol dimethacrylate (EGDMA) copolymers by initiated Chemical Vapor Deposition (iCVD): The working pressure was set to 800 mTorr, the filament was heated to 250 °C and the substrate was held at 30 °C. For all the samples, an initiator flow rate of 0.6 sccm of *tert*-butyl peroxide was used. The PFDA and EGDMA flow rates are stated in Table S1. The total flow rate was controlled to 4.8 sccm by adjusting the Nitrogen patch flow accordingly.

Table S1. Flow rates of the PFDA monomer (F_{PFDA}) and the crosslinker EGDMA (F_{EGDMA}) employed in the iCVD polymerization.

	F_{PFDA} [sccm]	F_{EGDMA} [sccm]
**p-PFDA**	0.15 ± 0.01	-
**23 % EGDMA**	0.17 ± 0.01	0.02 ± 0.02
**31 % EGDMA**	0.20 ± 0.01	0.05 ± 0.02
**51 % EGDMA**	0.16 ± 0.01	0.20 ± 0.02
**p-EGDMA**	-	0.10 ± 0.02

Atomic force microscopy

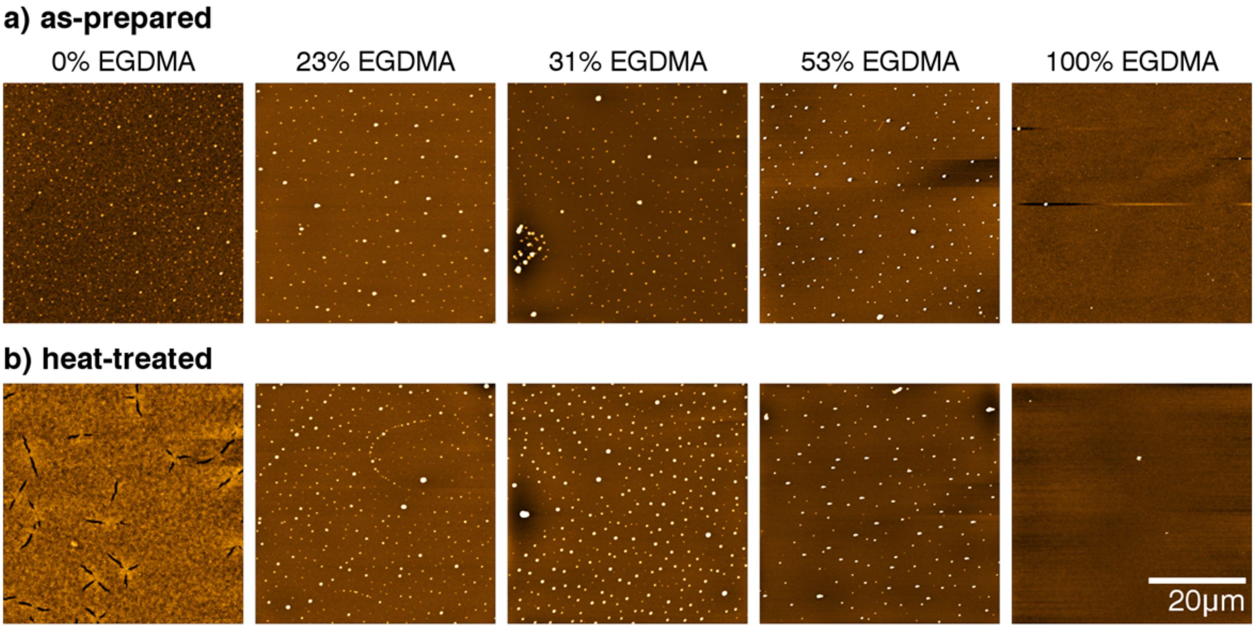

Figure S1. AFM micrographs of as-prepared (a) and heat-treated (b) p-PFDA films with different degrees of EGDMA cross-linking, depicting larger scales. The data are represented on individual color scales for clarity.

X-ray reflectivity measurements

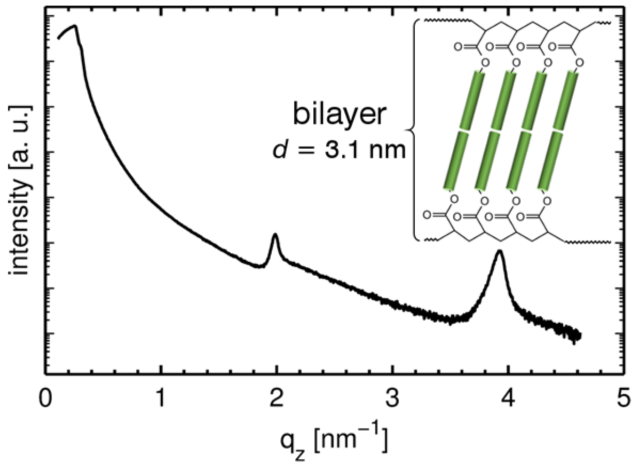

Figure S2. X-ray reflectivity data of an as-prepared p-PFDA film, exhibiting first and second order diffraction peaks corresponding to the bilayer packing of the perfluorinated groups. The inset shows a scheme of the p-PFDA lamellar structure, with the $CH_2CH_2(CF_2)_7CF_3$ groups being represented by cylindrical rods.
